# Introducing the Node Reporting and Data System 1.0 (Node-RADS): a concept for standardized assessment of lymph nodes in cancer

**DOI:** 10.1007/s00330-020-07572-4

**Published:** 2021-02-14

**Authors:** Fabian H. J. Elsholtz, Patrick Asbach, Matthias Haas, Minerva Becker, Regina G. H. Beets-Tan, Harriet C. Thoeny, Anwar R. Padhani, Bernd Hamm

**Affiliations:** 1 Department of Radiology, Charité – Universitätsmedizin Berlin, corporate member of Freie Universität Berlin, Humboldt-Universität zu Berlin, and Berlin Institute of Health, Charitéplatz 1, 10117 Berlin, Germany; 2 Division of Radiology, Department of Imaging and Medical Informatics, Geneva University Hospitals, University of Geneva, Geneva, Switzerland; 3 Department of Radiology, The Netherlands Cancer Institute, Amsterdam, The Netherlands; 4 Department of Diagnostic and Interventional Radiology, Fribourg Cantonal Hospital, Faculty of Medicine, University of Fribourg, Fribourg, Switzerland; 5 Paul Strickland Scanner Centre, Mount Vernon Cancer Centre, Northwood, UK

**Keywords:** Consensus, Lymph nodes, Magnetic resonance imaging, Neoplasms, Tomography, X-ray computed

## Abstract

**Abstract:**

“Node-RADS” addresses the lack of consensus in the radiologic assessment of lymph node involvement by cancer and meets the increasing demand for structured reporting on the likelihood of disease involvement. Node Reporting and Data System 1.0 (Node-RADS) systematically classifies the degree of suspicion of lymph node involvement based on the synthesis of established imaging findings. Straightforward definitions of imaging findings for two proposed scoring categories “size” and “configuration” are combined into assessment categories between 1 (“very low likelihood”) and 5 (“very high likelihood”). This scoring system is suitable for assessing likely involvement of lymph nodes on CT and MRI scans. It can be applied at any anatomical site, and to regional and non-regional lymph nodes in relation to a primary tumor location. Node-RADS will improve communication with referring physicians and promote the consistency of reporting for primary staging and in response assessment settings.

**Key Points:**

*• Node-RADS standardizes reporting of possible cancer involvement of regional and distant lymph nodes on CT and MRI.*

*• Node-RADS proposes the scoring categories “size” and “configuration” for assigning the 5-point Node-RADS score from 1 (“very low likelihood”) to 5 (“very high likelihood”).*

*• Node-RADS aims to increase consensus among radiologists for primary staging and in response assessment settings.*

**Supplementary Information:**

The online version contains supplementary material available at 10.1007/s00330-020-07572-4.

## Introduction

### Background

Evaluation of lymph nodes for the likelihood of disease involvement is important in the context of cancer staging because nodal involvement is a powerful adverse prognostic indicator that often determines patient management, frequently distinguishing surgical candidates from those best suited for non-surgical management [1, 2]. In most cases, the incidence of nodal involvement increases with tumor bulk and stage and is dependent on tumor histological type and grade.

Even though numerous studies have evaluated morphologic criteria in computed tomography (CT), magnetic resonance imaging (MRI), and ultrasound (US), practically there is still no consensus on which criteria should be used, although nodal size is generally accepted [3]. Paralleling approaches to assess size include short- and long-axis diameters, as well as volumetric measurements. However, lymph node size is a poor indicator for predicting the presence of secondary malignancy. In a study focusing on mesorectal lymph nodes in rectal cancer patients, Gina Brown and colleagues showed a broad size overlap between histologically benign (2–10 mm) and malignant (3–15 mm) lymph nodes [3]. Furthermore, using nodal size as a criterion for metastatic involvement can be confusing in the head and neck (HN) region as benign nodes have different sizes depending on patient age and on anatomic location; e.g., submandibular nodes are typically larger than lymph nodes in other neck groups (sometimes called stations/levels). The choice of the size cutoff will influence sensitivity and specificity for the detection of nodal metastases. In a HN study, Curtin and colleagues showed that a size cutoff of 10 mm in the largest axial nodal diameter results in a sensitivity and specificity of 88% and 39%, respectively, whereas a size cutoff of 15 mm yielded corresponding values of 56% and 84%, respectively [4]. Also, there is no consensus on whether lymph nodes should be measured in the axial or craniocaudal dimensions.

Criteria for lymph node configuration also exist with numerous descriptors, which can sometimes be helpful [5–8]. There have been some promising approaches by combining size and configuration criteria to facilitate and standardize the diagnostic workup for lymph nodes at specific anatomic locations, such as in the mesorectum in a European Society of Gastrointestinal and Abdominal Radiology (ESGAR) consensus statement [9]. However, focusing on specific disease entities and body sites limits the practical application of the approach in clinical practice.

In addition to being highly dependent on the interpretative skills of radiologists, unstructured reports that use a variety of terms to describe the likelihood of disease involvement also cause difficulties for clinical referrers making treatment decisions. Recently, there have been attempts at standardizing the reporting of oncological scans with the increasing adoption of the Reporting and Data Systems (RADS) for many scenarios for the detection and characterization of lesions within specific organs. For example, BI-RADS is well established for reporting on the detection of breast cancer using x-ray mammography [10]. Recently, other systems incorporating the -RADS acronym have emerged including LI-RADS for the MRI evaluation of hepatocellular carcinoma in liver cirrhosis, NI-RADS for surveillance of head and neck cancer, and PI-RADS for the MRI detection of prostate cancer, finding acceptance among radiologists and referring clinicians alike [10–13]. Structured reports can improve the reliability and validity of imaging assessments in the clinical routine but can also aid research studies and audits.

Generally, -RADS systems incorporate a Likert-type scale for the likelihood of the target disease being present. The use of scoring systems to assess the likelihood of malignant disease involvement has emerged, but is limited to specific body sites, cancer entities, time of evaluation, or imaging modalities [14–17]. There is no generally applicable imaging system for the whole body that indicates the scaled likelihood of malignant nodal disease involvement.

### Purpose

Node-RADS is a concept that addresses the aforementioned limitations, aiming to enhance the reporting of regional and distant lymph nodes in cancer patients by (i) clearly defining imaging criteria to increase consensus among radiologists, (ii) facilitating standardized lymph node reporting, and (iii) having broad applicability to cancer types across multiple anatomic sites, being evaluated by CT and/or MRI. To serve these purposes, the Node-RADS scheme is graphically depicted, is logically arranged, and is intuitive, enabling its adoption into the clinically routine without the need for additional time expenditure.

## Rationale and discussion of the system

### Overview and general considerations

**Fig. 1:**
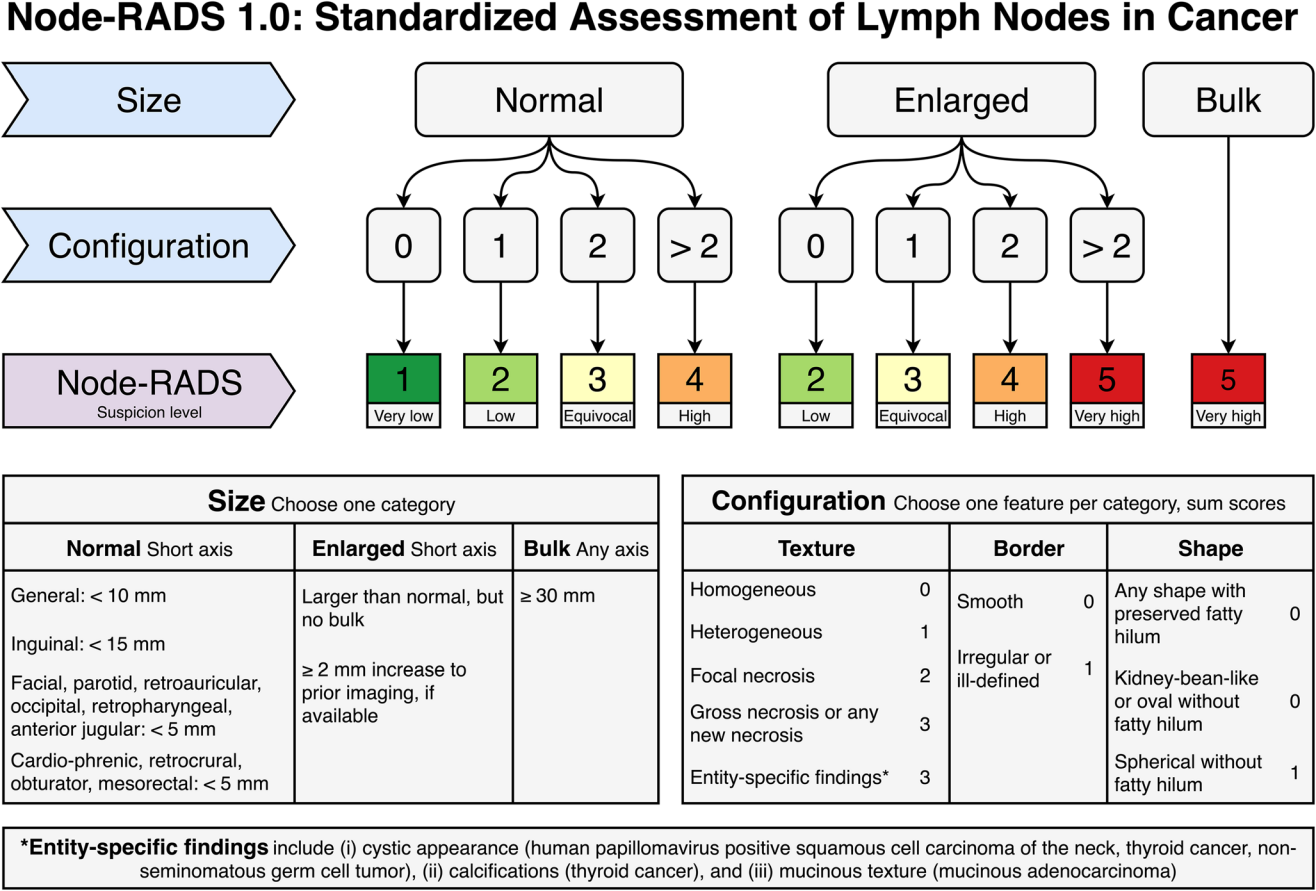
Node-RADS flowchart with a brief description of the criteria for lymph node assessment

The evaluation of a lymph node using the Node-RADS scheme results in an assessment category scored between 1 and 5, which reflects the level of suspicion for involvement by malignancy: “1—very low”; “2—low”; “3—equivocal”; “4—high”; “5—very high.” For this purpose, the interpreting radiologist is guided through a three-level flowchart (Fig. 1). Levels 1 and 2 address the two principle imaging criteria of “size” and “configuration.” Level 3 provides the resulting Node-RADS assessment score. Categories, definitions, and features of the respective criteria are also given in Fig. 1.

### Criterion “size”

There is an ongoing debate about the setting of limits for physiologic and pathologic size of lymph nodes. Ultimately, any chosen size cutoffs depend on the desired sensitivity and specificity concerning the stage of the primary tumor and/or clinical status of patients [18, 19]. Many radiologists do not consider nodal size to be an absolute criterion for the assessment of likely malignant involvement. However, since the size of lymph nodes is a criterion that is widely used, and is incorporated into the RECIST (Response Evaluation Criteria in Solid Tumors) criteria for response assessment studies [20], Node-RADS divides nodal sizes into three categories: “normal,” “enlarged,” and “bulk.”

In general, a lymph node is defined to have a “normal” size, when its short-axis diameter is < 10 mm. Node-RADS defines exceptions for normal size for inguinal nodes which could measure < 15 mm in short-axis diameter; on the other hand, Node-RADS sets a lower cutoff for specific subregions, where the normal short-axis diameter should be < 5 mm: facial, parotid, retroauricular, occipital, retropharyngeal, anterior jugular, retrocrural, cardio-phrenic, mesenteric, obturator, and mesorectal lymph nodes [20]. A “bulk” is defined as a lymph node with the longest diameter of ≥ 30 mm measured in any dimension. Since there are no generally accepted measurements for the term “bulk,” especially outside of lymphomas, we were guided by the TNM classification for head and neck cancer, where a lymph node diameter of 30 mm is the threshold for N2 (with the exception of HPV-positive oropharyngeal, nasopharyngeal, and thyroid cancer) [1]. Lymph nodes of “enlarged” size do not meet either the definitions of the categories “normal” or “bulk” or are between 10 and 15 mm (short axis) for RECIST purposes, or show interval increases of ≥ 2 mm in short-axis diameter, if prior imaging datasets are available for comparison. The ≥ 2-mm threshold is based on a consideration regarding the spatial resolution of CT scans (512 matrix over a 40–50-cm field-of-view results in a pixel size of 0.78 to 0.98 mm). The requirement of two pixels for a reliable measurement translates into axial dimensions of ≥ 2 mm.

Node-RADS seeks not to overinterpret the size of lymph nodes, which in a specific case means that an “enlarged” lymph node by size criteria without accompanying abnormal morphologic features discussed under the “configuration” criteria (below) will receive a Node-RADS score of 2 (“low suspicion”).

### Criterion “configuration”

After using the “size” criterion in the first-level evaluation, the second level considers other anatomic features under the criterion of “configuration” which have a critical importance for the final Node-RADS assessment score. The “configuration score” is made up of the summed numerical value from the three sub-categories of “texture,” “border,” and “shape.”

**Fig. 2:**
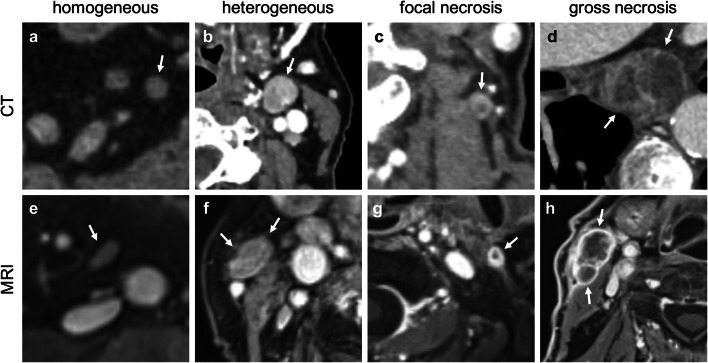
**a–h** Representative images for criterion “configuration,” feature “texture,” part 1 (homogeneous, heterogeneous, focal necrosis, gross necrosis). **a** Mesenteric, axial, contrast-enhanced (venous phase); **b** neck level IIa on the left side, axial plane, contrast-enhanced (split-bolus); **c** neck level V on the left side, axial, contrast-enhanced (split-bolus); **d** middle mediastinum, axial, contrast-enhanced (venous phase); **e** mesenteric, axial, T1 weighted, contrast-enhanced fat-suppressed; **f** parotid space on the right side, axial, T1 weighted, contrast-enhanced fat-suppressed; **g** parotid space on the left side, axial, T1 weighted, contrast-enhanced fat-suppressed; **h** parotid space on the right side, axial, T1 weighted, contrast-enhanced fat-suppressed

**Fig. 3:**
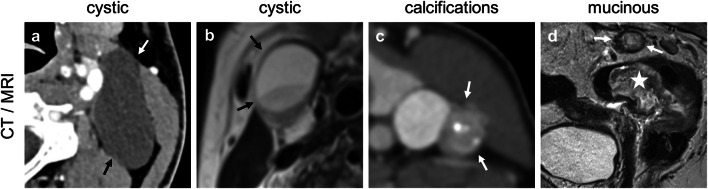
**a–d** Representative images for criterion “configuration,” feature “texture,” part 2 (entity-specific findings). **a** Neck level IIa on the left side, axial plane, contrast-enhanced (split-bolus); **b** neck level III on the right side, T2 weighted; **c** neck level IV on the left side, contrast-enhanced (venous phase); **d** mesorectal adjacent to a T3 mucinous type rectal adenocarcinoma (indicated by a white star), T2 weighted
- The category “border” evaluates possible extranodal disease extension. This is very specific in the histopathological sense, but often difficult to diagnose with imaging methods [23]. Therefore, either 0 points (“smooth”) or 1 point (“irregular or ill-defined”) contribute to the configuration score in this category (Fig. 4).

- The category “texture” refers to the internal structure of a lymph node, which is defined as “homogeneous” (0 points), “heterogeneous” (1 point), “focal necrosis” (2 points), and “gross necrosis or any new necrosis” (3 points). Also, 3 points can be assigned if other features summarized under “entity-specific findings” are present: (i) cystic appearance (human papillomavirus positive squamous cell carcinoma of the neck, thyroid cancer, and non-seminomatous germ cell tumor), (ii) calcifications (thyroid cancer), and (iii) mucinous texture (mucinous adenocarcinoma) [12, 21, 22] (Figs. 2 and 3). “Texture” seems to be the most reliably assignable category, which is why the highest numerical values can be achieved here compared with the other categories.

**Fig. 4:**
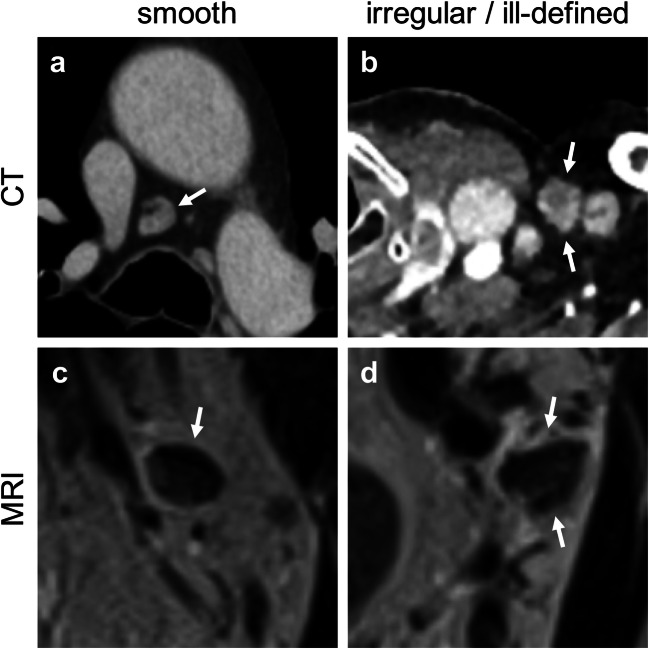
**a–d** Representative images for criterion “configuration,” feature “border.” **a** Middle mediastinum, axial, contrast-enhanced (venous phase); **b** medial supraclavicular on the left side, axial, contrast-enhanced (split-bolus), **c** obturator on the left side, coronal, T2 weighted; **d** obturator on the left side, coronal, T2 weighted
- The third category “shape” covers two features, the geometric shape and the delineation of the fatty hilum. Both features are usually well assessed on high-spatial resolution CT or MRI but are unspecific so that the maximum of 1 point can be assigned: 0 points (“any shape with preserved fatty hilum”) or (“kidney-bean-like or oval without fatty hilum”) 1 point (“spherical without fatty hilum”) (Fig. 5).

**Fig. 5:**
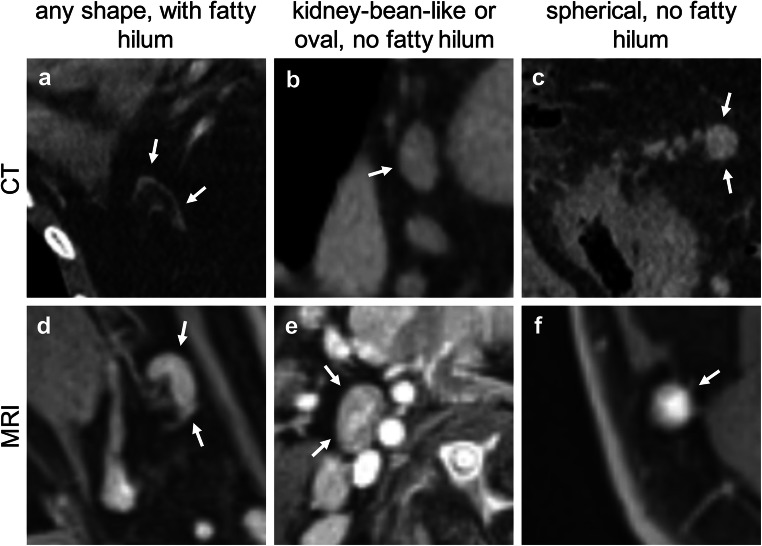
**a–f** Representative images for criterion “configuration,” feature “shape.” **a** Axillar on the left side, axial, contrast-enhanced (venous phase); **b** middle mediastinum, axial, contrast-enhanced (venous phase); **c** mesenteric on the right side, coronal, contrast-enhanced (venous phase); **d** neck level Ib on the left side, axial, T1 weighted, contrast-enhanced fat-suppressed; **e** neck level 2a on the right side, axial, T1 weighted, contrast-enhanced, fat-suppressed; **f** neck level V on the right side, T1 weighted, contrast-enhanced, fat-suppressed

One feature is chosen from each sub-category, which translates into a minimum achievable “configuration score” of 0 points, and a maximum possible score of 5 points.

As indicated in the Node-RADS flowchart (Fig. 1), each “configuration score” consecutively translates into the respective final Node-RADS score.

## Technical considerations

Node-RADS is applicable for the interpretation of lymph nodes on CT and MRI. Specific technical imaging acquisition parameters are not a prerequisite facilitating broad applicability of Node-RADS. The use of contrast material is not mandatory for MRI given the intrinsic high soft tissue contrast. The use of contrast material is mandatory for CT scans, to assess the “configuration” categories, in an appropriate parenchymal phase after intravenous injection.

For baseline staging, anatomic coverage should include all regional lymph nodes of the primary tumor, and, depending on the likelihood for distant metastatic disease, respective body parts (e.g., the chest and upper abdomen (adrenals) for staging HN tumors). For response assessment, anatomic coverage should include all lymph node sites that showed abnormal lymph nodes on baseline imaging.

## Structured reporting

To provide the best possible assessment of the nodal status of patients, radiologists are required to (a) know the purpose of the imaging study, i.e., staging at initial diagnosis, suspected recurrence or response assessment; (b) have detailed knowledge of tumor histology and stage of the primary tumor to determine the probability of nodal involvement; (c) have knowledge of the pattern of spread and the prevalence of micro- as opposed to macroscopic nodal involvement; (d) be familiar with the Node-RADS criteria for nodal involvement on MRI/CT at various anatomic sites and recognizing pitfalls in diagnosis; (e) have an idea of the accuracy of imaging observations and understand the impact of positive and negative imaging results on patient management; and (f) be familiar with new imaging methods for evaluating nodal disease including the value of PET/CT. In this context, Node-RADS is applicable to baseline staging and assessing the response to therapy. Representative cases for both applications are given in Figures 1 and 2 of the Electronic Supplementary Material.

### Staging objectives

- To identify the presence and extent of regional nodal metastases to assign an N-staging category.
- To identify whether the extent of nodal disease will significantly alter the surgical approaches. For example, by increasing the extent of surgical exploration, or the requirement for the placement of vascular grafts.
- To determine whether the presence of metastatic nodal involvement designates M-stage disease.
- To identify the presence and extent of regional nodal enlargement with a view to planning biopsy.
- To distinguish between nodal enlargement due to malignancy and that due to benign hyperplasia.
- To acknowledge the limitation of imaging to detect microscopic disease in normal size nodes (currently possible to some extent only with MR lymphography and PET-CT).

### Node reporting rules

- If there are multiple abnormal nodes in a specific nodal group, the node with the highest category should be reported with Node-RADS, unless the number of lymph node metastases influences TNM stage or treatment decision.
- In the context of TNM staging, Node-RADS 1 and 2 should be reported as N(−) and Node-RADS 4 and 5 as N(+). The decision how to report Node-RADS 3 should depend on the stage and histologic grade of the primary tumor (e.g., in Gleason 3 + 3 = 6 (UICC Grade 1) prostate cancer an obturator Node-RADS 3 lymph node should be reported as N(−), whereas, in adenocarcinoma of the pancreatic head, a peripancreatic Node-RADS 3 lymph node should be reported as N(+)). In the absence of histologic information, Node-RADS 3 should be reported as Nx.
- Although not required for TNM staging, it is sometimes necessary to classify in detail the sites of regional nodal involvement to facilitate surgical exploration (e.g., for head and neck tumors and in lung cancer—see relevant TNM/AJCC chapters for details) [1].
- There is often confusion about the precise anatomical location of nodal sites on cross-sectional imaging, particularly when planning radiotherapy or surgery. It is recommended that a standard nodal atlas is used depending on the use case.
- There is often confusion on nodal disease location at the border of regions. As a general rule, if a lymph node is located at the border of two regions, it should be assigned to the region in the direction of lymphatic drainage (e.g., common iliac as opposed to external iliac for prostate/bladder cancers).

### TNM staging

The TNM system places regional nodal involvement in the N category, but nodal involvement at other than regional sites is classified as distant metastases (i.e., belongs in the M category). It is therefore important for radiologists to know where regional and distant metastatic sites reside for each primary tumor. These details can be found in staging manuals, preferably in the AJCC Cancer Staging Manual [1]. Sometimes, the same organ may have different regional nodal groups; thus, the retroperitoneum is a distant metastatic site for cervical cancer but defined as “regional” for endometrial cancer.

It is important to note that the TNM system emphasizes different aspects for nodal involvement depending on the primary tumor; thus, for the bladder and head and neck cancers, nodal size is part of the N category. For many adenocarcinomas, the presence or absence of microscopic metastatic disease, regardless of primary tumor burden, is emphasized, whereas nodal involvement sometimes does not alter staging category at all (e.g., for well-differentiated follicular/papillary thyroid cancers in patients less than 45 years of age).

### RECIST reporting

The RECIST system is based on the measurement of target lesions, summing up lesion sizes to assess tumor response to therapy [20]. According to RECIST v1.1., lymph nodes should be measured along the short axis in the axial plane. To be considered pathologically enlarged and measurable, the short-axis diameter should be ≥ 15 mm. If the short-axis diameter is between 10 and 15 mm, they are considered pathologic, but cannot be used as target sites (non-measurable disease) [24].

In order to account for temporary nodal enlargement without clinical disease progression during immunotherapy, the RECIST system has been modified, so creating the iRECIST (immune-related RECIST) system [25].

The following general rules apply to Node-RADS regarding RECIST:

- Since Node-RADS incorporates the configuration criterion, nodes with a short-axis diameter < 15 mm or < 10 mm can be assigned a Node-RADS score of 3, 4, or 5 while being considered malignant non-measurable lesions or normal nodes, respectively, according to RECIST 1.1. These must not be regarded as contradictory, rather they reflect the different purposes of the two systems (detection versus response assessments).
- Since RECIST 1.1 is purely size-based, nodes with a short-axis diameter < 15 mm can be assigned a Node-RADS score of 2 while being considered pathologic according to RECIST 1.1. Again, this must not be regarded as contradictory, rather they reflect the different purposes of the two systems (see above).
- In the scenario when RECIST 1.1 and Node-RADS differ, this should trigger a careful evaluation of the respective node on follow-up imaging.
- Immunotherapy can induce reactive nodal enlargement and configuration changes that probably alter the Node-RADS score; therefore, iRECIST should be used in the context of likely pseudoprogression, and Node-RADS is not applicable here.

### Compliance with other RADS

Node-RADS can be used and reported in addition to already existing RADS (e.g., LI-RADS or PI-RADS). An exception is NI-RADS in the surveillance of head and neck cancer, where a score is already assigned for the cervical lymph nodes (“neck”). The criteria and algorithm of Node-RADS and NI-RADS are not concordant.

### Future directions

Undoubtedly, independent prospective studies on reliability (i.e., inter- and intra-reader agreement) and validity are mandatory to make any adjustments to the Node-RADS system proposed here. Although the combination of CT or MRI with functional sequences (e.g., perfusion imaging) or PET is widely used, these are intentionally not included in Node-RADS at this time to facilitate the straightforward use of the system. Ultrasound is frequently used for evaluation of superficial lymph nodes applying criteria that differ distinctly from CT and MRI and therefore not included in Node-RADS. However, as in existing -RADS, changes could expand the scope of the application upon the availability of further evidence [12]. Node-RADS is likely in need to be periodically modified in response to independent prospective studies that evaluate its efficacy, which may include the inclusion of additional imaging features, e.g., clustering of lymph nodes, or adjustments on the size cutoff for bulky disease. Finally, minimum technical imaging parameters might be a subject for inclusion in the standard in future iterations of Node-RADS.

## Conclusion

The Node Reporting and Data System 1.0 (Node-RADS) standardizes reporting of possible cancer involvement of regional and distant lymph nodes on CT and MR imaging. Node-RADS is applicable at any anatomical site, proposing the use in the scoring of the categories of “size” and “configuration” for assigning the 5-point Node-RADS assessment category score. An increase in the consensus of radiologic assessment of lymph nodes will facilitate uniformity of primary tumor staging, and evaluation of response to treatment.

## Supplementary Information

ESM 1 (DOCX 265 kb)

## Abbreviations

HN: Head and neck
MRI: Magnetic resonance imaging
NI: Neck imaging
RADS: Reporting and data system
TNM: Tumor, nodes, metastasis
