# Supplementary material for: Introducing the Node Reporting and Data System 1.0 (Node-RADS): a concept for standardized assessment of lymph nodes in cancer
Source: Eur Radiol. 2021 Feb 14;31(8):6116–24. doi: 10.1007/s00330-020-07572-4 (PMC8270876; doi:10.1007/s00330-020-07572-4)
Supplement: Supplementary file 1 — (DOCX 265 kb) [file 330_2020_7572_MOESM1_ESM.docx]

**Supplementary Material**

**Supplementary Figure 1**


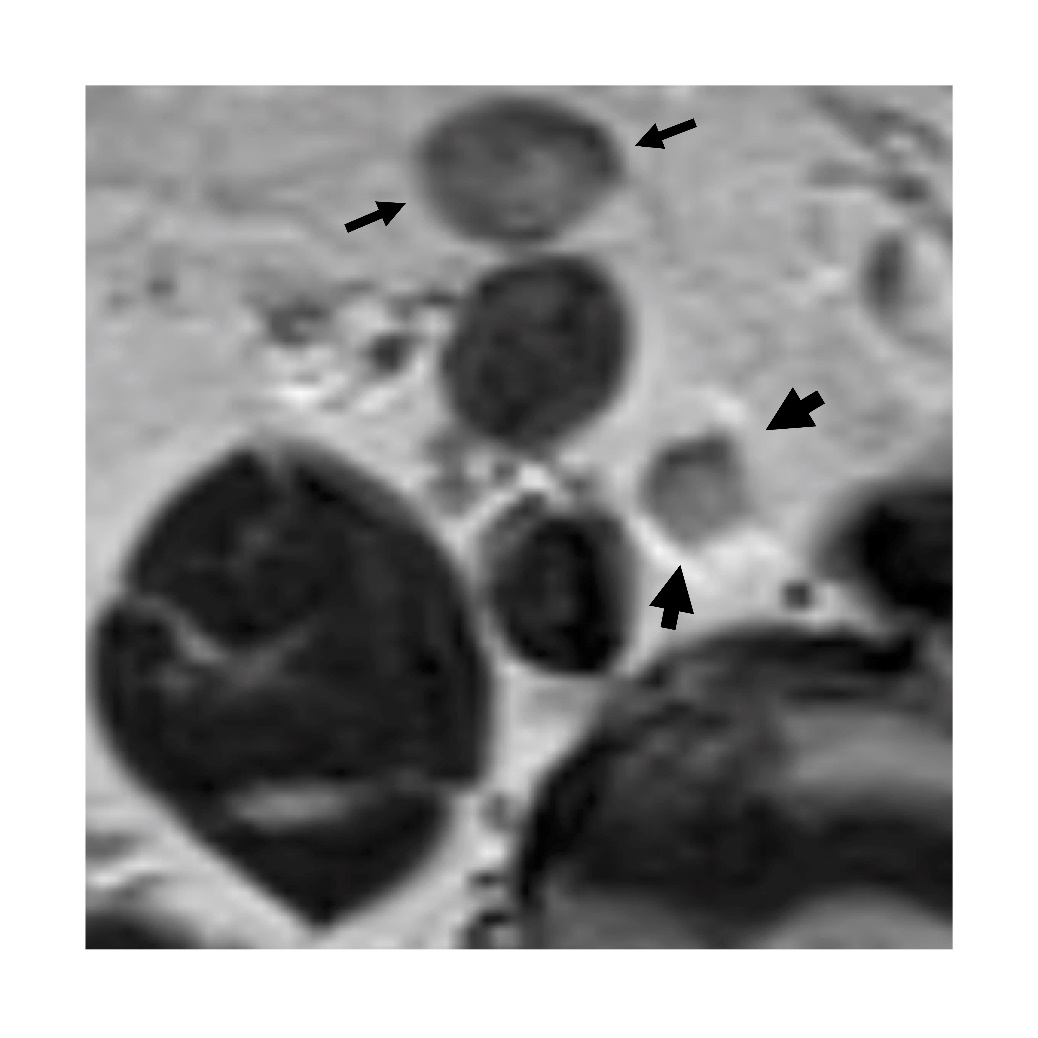


**Figure 1.** Representative case for Node-RADS assessment before therapy. 72 y/o old male with a neuroendocrine differentiated prostate cancer. Staging MRI (axial, T2 weighted) shows two right common iliac lymph nodes. The node anterior to the right common iliac artery (thin arrows) is assigned with a final Node-RADS assessment score of 3 (equivocal suspicion): 13 mm short axis diameter (enlarged size), heterogeneous texture (1 point), smooth border (0 points), oval shape (size 18 x 13 mm) without fatty hilum (0 points). The node medial to the right common iliac artery (thick arrows) is assigned with a final Node-RADS assessment score of 4 (high suspicion): 8 mm short axis diameter (normal size), heterogeneous texture (1 point), ill-defined border (1 points), spherical shape (size 8 x 8 mm) without fatty hilum (1 point). Since the node with the highest category should be reported with Node-RADS, a Node-RADS score of 4 is assigned in this case.

**Supplementary Figure 2**


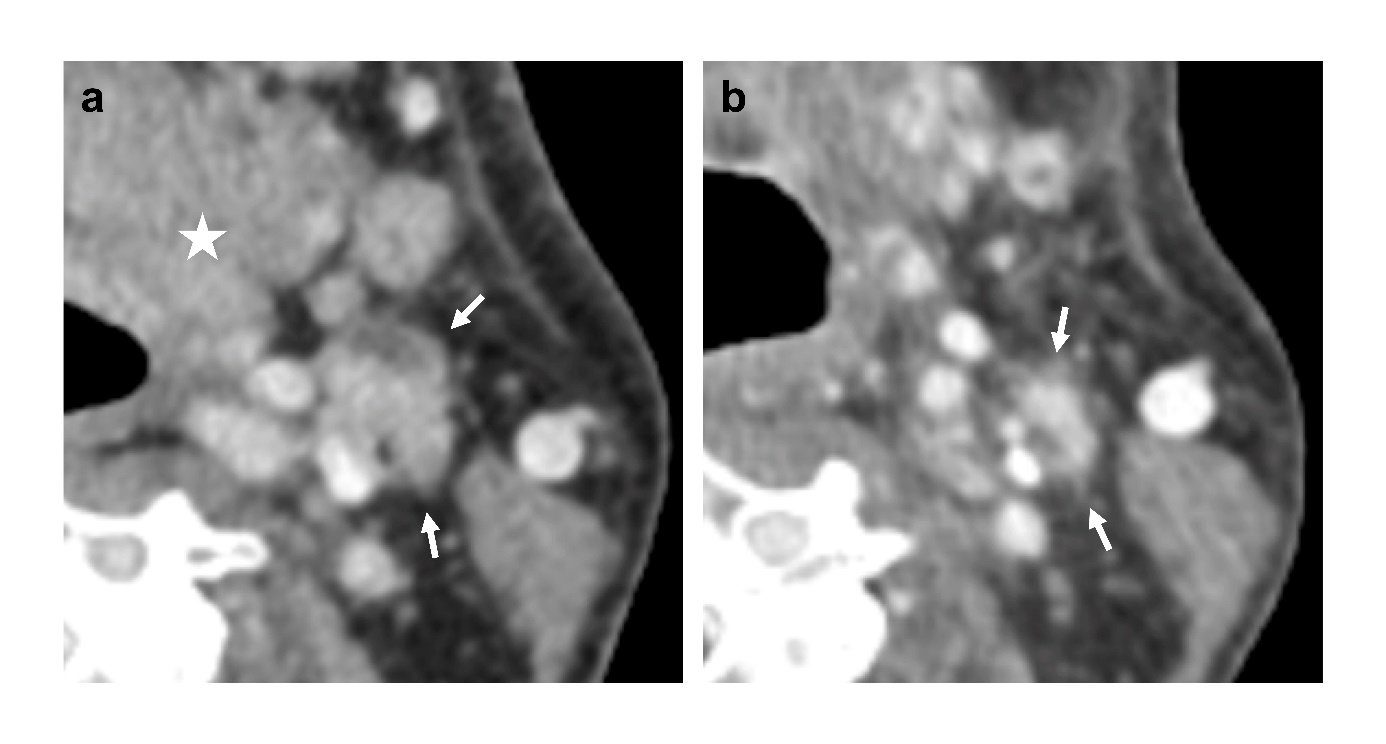


**Figure 2.** Representative case for Node-RADS assessment before and after therapy. 52 y/o male with a T4 squamous cell carcinoma of the left mouth floor (indicated by a white star). Pretreatment CT (a, axial, contrast-enhanced (split bolus)) shows a lymph node in level IIa on the left side receiving a final Node-RADS score of 5 (very high suspicion): 12 mm short axis diameter (enlarged size), focal necrosis (2 points), irregular border (1 point), polygonal shape (0 points). In the post-treatment CT (b, axial, contrast-enhanced (split bolus)), 4 months after definitive combined chemo-radiotherapy, the same lymph node is assigned with a Node-RADS score of 2 (low suspicion): 5 mm short axis diameter (normal size), homogeneous texture (0 points), ill-defined border (1 point), kidney-bean-like shape (0 points).
